# Resveratrol facilitates bone formation in high-glucose conditions

**DOI:** 10.3389/fphys.2024.1347756

**Published:** 2024-04-19

**Authors:** Sung-Min Hwang, Tae-Young Kim, Anna Kim, Yong-Gun Kim, Jin-Woo Park, Jae-Mok Lee, Jae-Young Kim, Jo-Young Suh

**Affiliations:** ^1^ Department of Periodontology, School of Dentistry, IHBR, Kyungpook National University, Daegu, Republic of Korea; ^2^ Department of Biochemistry, School of Dentistry, IHBR, Kyungpook National University, Daegu, Republic of Korea

**Keywords:** periodontitis, diabetes mellitus, periodontium, osteoblast, osteogenesis, differentiation

## Abstract

Periodontitis is known to be affected by high-glucose conditions, which poses a challenge to periodontal tissue regeneration, particularly in bone formation. In this study, the potential effects of resveratrol (3,5,4′-trihydroxystilbene, RSV) in facilitating bone formation under high-glucose conditions after periodontitis has been investigated. We focused on the analysis of osteoblasts and periodontal ligament cells, which are essential for bone formation including cell proliferation and differentiation. And we aimed to investigate the impact of RSV on bone healing, employed diabetic mouse model induced by streptozotocin and confirmed through histological observation. High-glucose conditions adversely affected cell proliferation and ALP activity in both MC3T3-E1 and hPDLF *in vitro*, with more significant impact on MC3T3-E1 cells. RSV under high-glucose conditions had positive effects on both, showing early-stage effects for MC3T3-E1 cells and later-stage effects for hPDLF cells. RSV seemed to have a more pronounced rescuing role in MC3T3-E1 cells. Increased ALP activity was observed and the expression levels of significant genes, such as Col 1, TGF-β1, ALP, and OC, in osteogenic differentiation were exhibited stage-specific expression patterns. Upregulated Col 1 and TGF-β1 were detected in the early stage, and then ALP and OC expressions became more pronounced in the later stages. Similarly, stronger positive reactions against RUNX2 were detected in the RSV-treated group compared to the control. Furthermore, in *in vivo* experiment, RSV stimulates the growth and differentiation of osteoblasts, thereby promoting bone formation. High-glucose levels have the potential to impair cellular functions and the regenerative capacity to facilitate bone formation with MC3T3-E1 rather than hPDLF cells. Resveratrol appears to facilitate the inherent abilities of MC3T3-E1 cells compared with hPDLF cells, indicating its potential capacity to restore functionality during periodontal regeneration.

## Introduction

Periodontitis, a serious oral disease, involves the progressive destruction of connective tissue and alveolar bone support in the periodontium (Genco and Borgnakke, 2000; Wu et al., 2015). It is highly prevalent in the United States, affecting around 45.9% of adults aged 30 years and older (Eke et al., 2015), causing negative effects on individuals’ overall wellbeing and systemic health (Reynolds and Duane, 2018; O Dowd et al., 2010). The long-term consequences of periodontitis manifest as tooth movement, drifting, teeth spacing, and gingival recession (Preshaw and Bissett, 2013). Periodontitis also damages tooth-supporting structures, leading to tooth mobility, which necessitates interventions such as dental implants. Therefore, treating periodontitis has always been our ongoing challenge. Unfortunately, the treatment outcomes significantly vary due to the influence of several factors, such as smoking, obesity, and systemic conditions (Bartold, 2000; Reynolds and Duane, 2018). These risk factors have long been considered to be biologically associated with diabetes mellitus (DM) and periodontitis (Bullon et al., 2000; Bell and Polonsky, 2001; Tsai et al., 2002; Wu et al., 2015). Several cross-sectional and longitudinal studies have consistently demonstrated that individuals with DM are at an increased risk of developing periodontitis, with approximately 3-4 times greater susceptibility than nondiabetic individuals (Preshaw and Bissett, 2013). A comprehensive analysis has shown a noticeable mutual association between DM and periodontitis (Wu et al., 2020).

DM, one of the systemic risk factors associated with periodontitis, is a medical condition marked by high blood glucose levels (Genco and Borgnakke, 2000; Wu et al., 2015). It adversely affects osteoblast and PDL fibroblast, both known to play an important role in bone tissue regeneration (Lozano et al., 2009; Wu et al., 2015). Previous studies also reported that osteoblast and PDL fibroblast are crucial cell sources responsible for alveolar bone restoration (Kim et al., 2020; Min et al., 2020; Bae et al., 2022). Furthermore, the absence of tissue repair observed in periodontal disease suggests that osteoblasts and PDL fibroblasts are either dysfunctional or unable to maintain tissue balance due to premature cell death or apoptosis (He et al., 2004; Liu et al., 2004). Existing evidence suggests that DM exacerbates osteoblast cell apoptosis in periodontitis, resulting in reduced bone integration (Pacios et al., 2013). DM also contributes to apoptosis by inducing reactive oxygen species (ROS) production, driven by inflammation and elevated glucose levels (Wu et al., 2015). ROS accumulation, associated with DM complications, leads to increased oxidative stress within the periodontal tissue, triggering osteoblast apoptosis. In particular, the role of ROS extends beyond periodontitis, as it is considered to play an important role in the progression of other inflammatory disorders (Correa et al., 2019). Intracellular ROS production is highly involved in bone homeostasis by intervening osteoblast differentiation (Tao et al., 2020). Furthermore, DM interferes with bone formation by reducing the expression of transcription factors that regulate osteoblast differentiation and decreases the population of PDL fibroblast by increasing their apoptotic rate (Isaka et al., 2001; Lu et al., 2003; Liu et al., 2006). This suggests that DM worsens periodontitis and accelerates bone resorption commonly associated with the disease (Wu et al., 2015). Therefore, the therapeutic objective is to restore lost periodontium resulting from diabetes-related periodontitis. To expedite the healing process and promote regeneration, researchers have conducted numerous studies on the use of bioactive materials.

Resveratrol (RSV; 3,5,4′-trihydroxystilbene), a polyphenolic phytoalexin present in different plants and fruits, has exerted positive effects in various diabetes-related pathological conditions (Turan et al., 2012; Xu et al., 2014; Bastianetto et al., 2015; Vella et al., 2015). It is believed to act as a potent antioxidant, reducing oxidative stress in diabetic tissues (Su et al., 2006). The beneficial effects of RSV have been attributed to its antioxidant and free radical scavenging properties (Leonard et al., 2003; Brasnyo et al., 2011). Several studies have investigated the impact of RSV on periodontium. RSV, as a Sirt1 activator, has been shown to inhibit apoptosis induced by hydrogen peroxide in MC3T3-E1 cells, which are involved in bone formation (He et al., 2015). In addition, it has been demonstrated to enhance the growth and maturation of mouse osteoblastic MC3T3-E1 cells (Mizutani et al., 1998; Dai et al., 2007). A recent study has shown the antioxidant and anti-inflammatory properties of RSV, which effectively mitigated alveolar bone loss caused by periodontitis (Bhattarai et al., 2016a; Min et al., 2020). These findings indicate that RSV holds promise as a potential therapeutic agent for managing and preserving the health of alveolar bone in individuals with periodontal diseases including periodontitis.

In this study, although RSV shows potential as a prospective therapeutic option for regenerating compromised tooth-supporting tissues, even in diabetes-related pathological conditions, its impact on cellular homeostasis remains unclear. Thus, it is important to understand how RSV influence differentiation promotion in osteoblasts and PDL fibroblasts, given their potential significance in maintaining cellular physiology. This study aimed to investigate these crucial aspects, unveiling the potential effects of RSV at the cellular level. Furthermore, an experimentally-induced periodontitis and streptozotocin-induced DM mouse model were employed to investigate the effects of RSV on the bone-healing process of extractions sockets.

## Materials and methods

### *In vitro* cultivation of MC3T3-E1 and human PDLF cells

The MC3T3-E1 cells used in this study were obtained from the American Type Culture Collection (CRL-2593; Manassas, VA, United States). These cells were isolated from the calvaria of C57BL/6 mice. The cells were cultured in alpha-minimum essential media (α-MEM; HyClone, Logan, UT, United States) and then supplemented with 10% fetal bovine serum (FBS) and antibiotics (100-U/mL penicillin and 100-U/mL streptomycin). Then, they were maintained in a controlled environment at 37°C with 5% CO_2_ and a humidified atmosphere. PDL tissues were obtained from extracted premolars (periodontally health and non-carious human teeth) of three female donors aged 10–20 years for orthodontic reasons at the Department of Periodontology, Kyungpook National University Hospital. The culture of human PDL fibroblasts (hPDLF) was isolated from these PDL cells according to a previously established protocol (Kim et al., 2006). This study was approved by the Institutional Review Board of the Department of Periodontology, Kyungpook National University Hospital (KNUH-74005-745), and we obtained informed consent from all the study participants and the legal guardians of those under the age of 18. The hPDLF cells were cultured in Dulbecco’s Modified Eagle’s Medium (DMEM) supplemented with normal glucose (1,100 mg/L; HyClone, Logan, UT, United States), 10% FBS (HyClone, Logan, UT, United States), and antibiotics (100-U/mL penicillin and 100-U/mL streptomycin (HyClone, Logan, UT, United States). The hPDLF cells between 4th to 7th passages were used in this study. To maintain optimal cell growth and viability, the culture mediums were refreshed every 3 days.

### Cell proliferation assay

The experimental procedures encompassed two main aspects: assessment of cell proliferation and investigation of the impact of various RSV concentrations both under high-glucose conditions. For these aspects, MC3T3-E1 and hPDLF cells were evaluated via CCK-8 assay for assessing of cell toxicity and cell survival rate using the EZ-Cytox Enhanced Cell Viability Assay Kit (DOGEN, Seoul, Korea) under high-glucose conditions according to the manufacturer instructions. The MC3T3-E1 cells were seeded into 96-well plates at a density of 5 × 10^3^ cells/well with α-MEM supplemented with 10% FBS, whereas the hPDLF cells were seeded into 96-well plates at a density of 1 × 10^4^ cells/well in low-glucose DMEM supplemented with 10% FBS. After 24 h of growth, the cells were starved using a serum-free medium containing 0.5% FBS for an additional 24 h. Subsequently, six divided cell flasks were used for one control group (normal glucose group) and five test groups (high-glucose with resveratrol concentration dependent groups): normal glucose (NG) group, cells cultured in normal glucose (1,100 mg/L or 5.5 mM) supplemented with 2% FBS, and high-glucose groups, cells cultured in high glucose (4,500 mg/L or 25.5 mM) supplemented with 2% FBS. In the high-glucose groups, the cells were exposed to a medium supplemented with 2% FBS, with high-glucose concentration (4,500 mg/L or 25.5 mM). RSV was added to the medium at different concentrations (0, 3.12, 6.25, 12.5, and 25 μM). To facilitate classification and ease of reference, the groups were labeled with the abbreviations (see Supplementary Material S1). The cells were incubated in their respective media for 1, 3, 5, and 7 days, as specified in previous studies [(Hamann et al., 2012; Kato et al., 2016; Dong et al., 2017)]. At each time point, 10 μL of CCK-8 solution was added to each well and incubated for 40 min at 37°C. Then, absorbance at 450 nm was measured using a microtiter plate enzyme-linked immunosorbent assay (ELISA) reader (BMG LABTECH, FLUOstar OPTIMA, Offenburg, Germany). Cell proliferation was calculated as a percentage relative to the control cells. The experiments were conducted independently and repeated three times to ensure the reliability and reproducibility of the results.

### Alkaline phosphatase activity

The effects of glucose conditions on the alkaline phosphatase (ALP) activity of MC3T3-E1 and hPDLF cells were evaluated. Both cells were seeded into 24-well plate at a density of 2 × 10^4^ cells/well in normal culture medium. After culture for 3 days until confluence, the time point was designated as day 0 for subsequent analysis. During the incubation period, the cells were treated with a specialized medium containing 2% FBS, 50-μg/mL ascorbic acid (Sigma-Aldrich, St. Louis, MO, United States), 10-mM beta-glycerophosphate (Sigma-Aldrich, St. Louis, MO, United States), and 100-nM dexamethasone (Sigma-Aldrich, St. Louis, MO, United States). The medium was prepared with two different glucose concentrations: NG (1,100 mg/L or 5.5 mM) and high glucose (4,500 mg/L or 25.5 mM). The cells were incubated in these respective media for 7 and 14 days. Also, for the high-glucose groups (MC3T3-E1 cell group), the incubation medium was supplemented with various RSV concentrations (0, 3.12, 6.25, 12.5, and 25 μM). The cells were incubated in these respective media for 7 and 14 days. An ALP assay kit (AnaSpec, Fremont, CA, United States) was used to assess the ALP activity. The supernatants from each experimental group were collected, and the enzyme activity was standardized by normalizing to the protein concentration of the cells. Furthermore, the supernatants were combined with 50-μL p-nitrophenylphosphate substrate solution and incubated at 37°C for 45 min to assess the ALP activity. The reaction was halted by the addition of 50-μL stop solution. Absorbance of the samples was measured at 405 nm using a microtiter plate ELISA reader. Standard curve determination and sample evaluation were performed according to the manufacturer’s instructions. The experiments were independently conducted in triplicate to ensure the accuracy and reproducibility of the results.

### Real-time polymerase chain reaction (RT-PCR)

The MC3T3-E1 cells were separately seeded into a 6-well plate at a density of 1 × 10^5^ cells/well. After 24 h of incubation in normal culture medium, the cells reached confluence, which was designated as day 0 (Supplementary Material S2). Subsequently, the cells were treated with a medium supplemented with 2% FBS, 50-µg/mL ascorbic acid, 10-mM beta-glycerophosphate, and 100-nM dexamethasone, containing either high glucose (4,500 mg/L or 25.5 mM) or RSV (25 μM) with high glucose. Total RNA was extracted from the cells on days 1, 7, 14, and 21 using TRIzol reagent (Invitrogen, Grand Island, NY, United States). Then, 2 µg of total RNA was used to synthesize cDNA using the iScript™ cDNA Synthesis Kit (Bio-Rad Laboratories, Inc., Hercules, CA, United States). Real-time polymerase chain reaction (RT-PCR) was performed using iQ SYBR Green Supermix (Bio-Rad Laboratories, Inc., Hercules, CA, United States) to measure the gene expression levels of ALP, collagen type 1 (Col 1), transforming growth factor-beta 1 (TGF-β1), osteocalcin (OC), and an endogenous control gene (glyceraldehyde-3-phosphate dehydrogenase, GAPDH) with a specified thermal cycler (Bio-Rad Laboratories, Inc., Hercules, CA, United States). For the amplification, 35 cycles were performed under the following temperature and time conditions: 95°C for 20 s, 58°C–60°C for 25 s, and 72°C for 1 min. These conditions were applied with the specific primers (Table 1). Each sample was analyzed in triplicate, and the resulting data were normalized to the expression level of the housekeeping gene GAPDH. The overall *in vitro* experimental design is presented as a schematic diagram in Supplementary Material S2.

**TABLE 1:** List of primers used for RT-PCR analysis of MC3T3-E1 cells and hPDLF cells F: Forward, R: Reverse, ALP: Alkaline phosphatase, TGF-β1: Transforming growth factor- β1, Col 1: Collagen type 1, OC: Osteocalcin, GADPH: Glyceraldehyde-3 phosphate dehydrogenase.

Gene	Primer sequence for MC3T3-E1 cells	Gene accession number
ALP	F: 5′-ATC​TTT​GGT​CTG​GCT​CCC​ATG-3′	NM_007431.3
	R: 5′-TTT​CCC​GTT​CAC​CGT​CCA​C-3′	
Col 1	F: 5′-AAC​AGT​CGC​TTC​ACC​TAC​AGC-3′	NM_007742.4
	R: 5′-GGT​CTT​GGT​GGT​TTT​GTA​TTC​G-3′	
TGF-β1	F: 5′-CAA​CCC​AGG​TCC​TTC​CTA​AA-3′	NM_011577.2
	R: 5′-GGA​GAG​CCC​TGG​ATA​CCA​AC-3′	
OC	F: 5′-CCA​CAG​CCT​TCA​TGT​CCA​AG-3′	NM_001032298.3
	R: 5′-GGC​AGA​GAG​AGA​GGA​CAG​GG-3′	
GAPDH	F: 5′-GGT​GCT​GAG​TAT​GTC​GTG​GA-3′	
	R: 5′-CAG​TTG​GTG​GTG​CAG​GAT​G-3′	

Gene	Primer sequence for hPDLF cells	Gene accession number
ALP	F: 5′ -CTG​CCA​TCC​TGT​ATG​GCA​ATG-3′	NM_000478.6
	R: 5′ -AGA​CTG​CGC​CTG​TAG​TTG​TTG-3′	
Col 1	F: 5′ -AGA​CGA​AGA​CAT​CCC​ACC​AAT​C-3′	NM_000088.4
	R: 5′ -GAT​CAC​GTC​ATC​GCA​CAA​CAC-3′	
TGF-β1	F: 5′ -GGA​TAC​CAA​CTA​TTG​CTT​CAG​CTC​C-3′	NM_000660.7
	R: 5′ -AGG​CTC​CAA​ATG​TAG​GGG​CAG​GGC​C-3′	
OC	F: 5′ -TAG​TGA​AGA​GAC​CCA​GGC​GCT​A-3′	NM_199173.6
	R: 5′ -TCA​CAG​TCC​GGA​TTG​AGC​TCA-3′	
GAPDH	F: 5′ -CCG​CAT​CTT​CTT​TTG​CGT-3′	
	R: 5′-AGT​TAA​AAG​CAG​CCC​TGG​TG-3′	

### Experimentally induced periodontitis and diabetes mellitus mouse model

Male C57BL/6 mice, aged six-week-old, were used in the study. They were housed under controlled conditions following previous study (Min et al., 2020; Bae et al., 2022). Following a 5-day adaptation period, diabetes was induced in all mice using streptozotocin (STZ, Sigma-Aldrich, St. Louis, MO, United States). The induction procedure involved administering multiple low doses of STZ (40 mg/kg, intraperitoneally; i.p.) for 5 consecutive days with the following procedure (Furman, 2015). After a period of 4 weeks following STZ administration, blood glucose levels were measured in tail-vein blood samples with a blood glucose measuring device (ACCU-CHEK Performa^®^, Roche Diagnostics, Basel, Switzerland). Mice with fasting glucose levels above 300 mg/dL were classified as having induced diabetes (Furman, 2015). To investigate the impact of DM on bone-healing capacity, the mice induced with DM were compared previous study with mice in normal glucose levels (see Supplementary Material S3). And then, assessment of the bone-healing capacity of RSV in high-glucose conditions with mouse model was employed. The mice in the study were assigned two groups (20 mice in total): control group (high-glucose group; 10 mice per group) and experimental group (RSV with high-glucose group; 10 mice per group). To induce periodontitis, the maxillary right second molars of all mice were ligated with 5-0 black silk (SK54510, AILEE CO., LTD. Busan, Korea) for a period of 5 days under anesthesia, following a previously established protocol (Adhikari et al., 2019). The care, maintenance, and treatment of the animals in this study strictly adhered to the guidelines of the Intramural Animal Use and Care Committee of Kyungpook National University, School of Dentistry (KNU 2015-136). After 5 days of ligature, the maxillary right second molars were extracted under anesthesia using an explorer. In the control group, the extraction socket was treated with 2 μL of Pluronic^®^ F-127 (Sigma-Aldrich, Munich, Germany) and 0.01% dimethyl sulfoxide (DMSO, Duchefa Biochemie, Harlem, Netherlands). In the experimental group, a mixture containing RSV, Pluronic^®^ F-127% and 0.01% DMSO was applied to the socket. The final concentration of RSV in the mixture was maintained at 25 μM. The delivery of the drug into the socket was carried out using a gas-tight Hamilton syringe (Hamilton, Reno, NV, United States) as previously described in a published study (Adhikari et al., 2019). After application, the wound was sealed using a fibrin sealant (Tisseel; Baxter, Deerfield, IL, United States). The mice were sacrificed at various time points (days 7, and 14) after the application of RSV or vehicle in the socket for further analysis.

### Histology and immunohistochemistry

For histological analysis, mouse maxillary bones were excised and fixed in a solution of 4% paraformaldehyde in phosphate-buffered saline (PBS). The specimens were then kept at a temperature of 4°C overnight to ensure proper fixation. They were rinsed twice with PBS and placed in an ethylenediaminetetraacetic acid (EDTA) solution at a temperature of 4°C for a period of 4 weeks to undergo decalcification. To ensure effective decalcification, the EDTA solution was refreshed every 2 days. Once decalcified, the fixed specimens underwent dehydration using ethanol and xylene. They were then embedded in paraffin. The decalcified specimens were sectioned into slices with a thickness of 7 μm. These slices were carefully mounted on slides that had been coated with poly-l-lysine (Muto Pure Chemicals, Tokyo, Japan) to improve the adherence of tissue sections. After fixation and decalcification process, the harvested maxillae were carefully dissected along the palatal rugae, ensuring that both the right and left maxillary second molars were positioned together on a single slide. This approach facilitated the examination and comparison of the adjacent teeth during the subsequent histological analysis. For histological examination, Masson’s trichrome (MTC) stain was used to assess the structural orientation and integration of the PDL with bone and cementum. These staining provided valuable insight into the tissue morphology and composition, offering insights into the structural organization and connectivity of various tissue components within the periodontium.

Immunohistochemistry was also performed previously described (Bae et al., 2022). Primary antibodies against runt-related transcription factor 2 (RUNX2; cat. No. ab192256; 1:1000; Abcam, United Kingdom) were used to investigate bone formation and differentiation. Secondary antibodies were used as biotinylated anti-rabbit or anti-rat IgG. Binding of the primary antibody to the sections was visualized using the diaminobenzidine tetrahydrochloride reagent kit (GBI Labs, Bothell, WA, United States, cat no. C09-12).

### Image analysis

All slides prepared for histology and immunohistochemistry were examined using a DM2500 microscope (Leica Microsystems, Wetzlar, Germany) equipped with a DFC310 FX digital CCD camera (Leica Microsystems). ImageJ software was utilized for the statistical analysis of bone formation levels in the specified regions of Masson’s trichrome (MTC) stained tissues, following established protocol (Adhikari et al., 2019). In brief, the original MTC stained images were converted to RGB format and then deconvolved using the color deconvolution plugin in ImageJ. Subsequently, after setting a consistent threshold, the area and integrated density of collagen fibers within the region of interest were quantified. For this analysis, six different images covering an area of 200 μm^2^ were analyzed from five randomly selected specimens. Additionally, the quantification of RUNX2 expression involved counting the number of positive cells in immunostained sections. This was performed using six images covering an area of 100 μm^2^ each, obtained from five different specimens.

### Statistical analysis

Statistical analysis was conducted using the SPSS software version 21 (IBM Corp., Chicago, IL, United States). Data obtained from this study were analyzed via one-way analysis of variance to determine significant differences between the groups. *Post hoc* analysis was conducted using Tukey’s honestly significant difference test to compare the means between the groups. The results were expressed as mean ± standard deviation, and repeated three times of experiment to ensure the reliability and consistency of the results. *p* < 0.05 was considered to indicate statistical significance.

## Results

### Cell proliferation and ALP activity under high-glucose conditions

**FIGURE 1:**
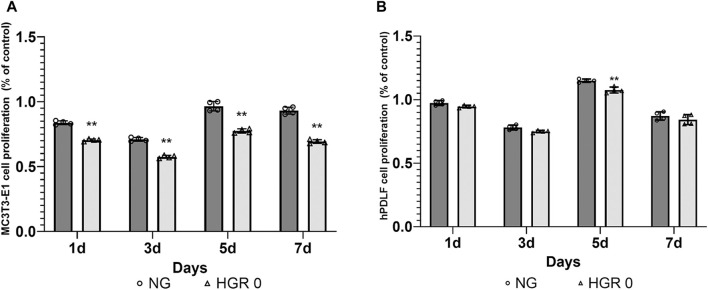
Effect of high-glucose on the proliferation of MC3T3-E1 **(A)** and hPDLF cells **(B)**. The effect of high glucose on cell proliferation was investigated via CCK-8 assay at different time points (1, 3, 5, and 7 days). For MC3T3-E1 cells, the proliferation rate was significantly reduced by high glucose at all time points (*p <* 0.01) **(A)**. The results indicated significant decrease in the rate of cell proliferation for hPDLF cells in the high-glucose (HGR 0) group compared with the normal glucose (NG) group specifically on day 5 (*p <* 0.01) **(B)**. This suggests that MC3T3-E1 cells are more adversely affected by high glucose than hPDLF cells. The experiments were conducted independently in triplicate. *: Statistically significant differences compared with the NG group at each time point (*p* < 0.05). **: Statistically significant differences compared with the NG group at each time point (*p* < 0.01).

**FIGURE 2:**
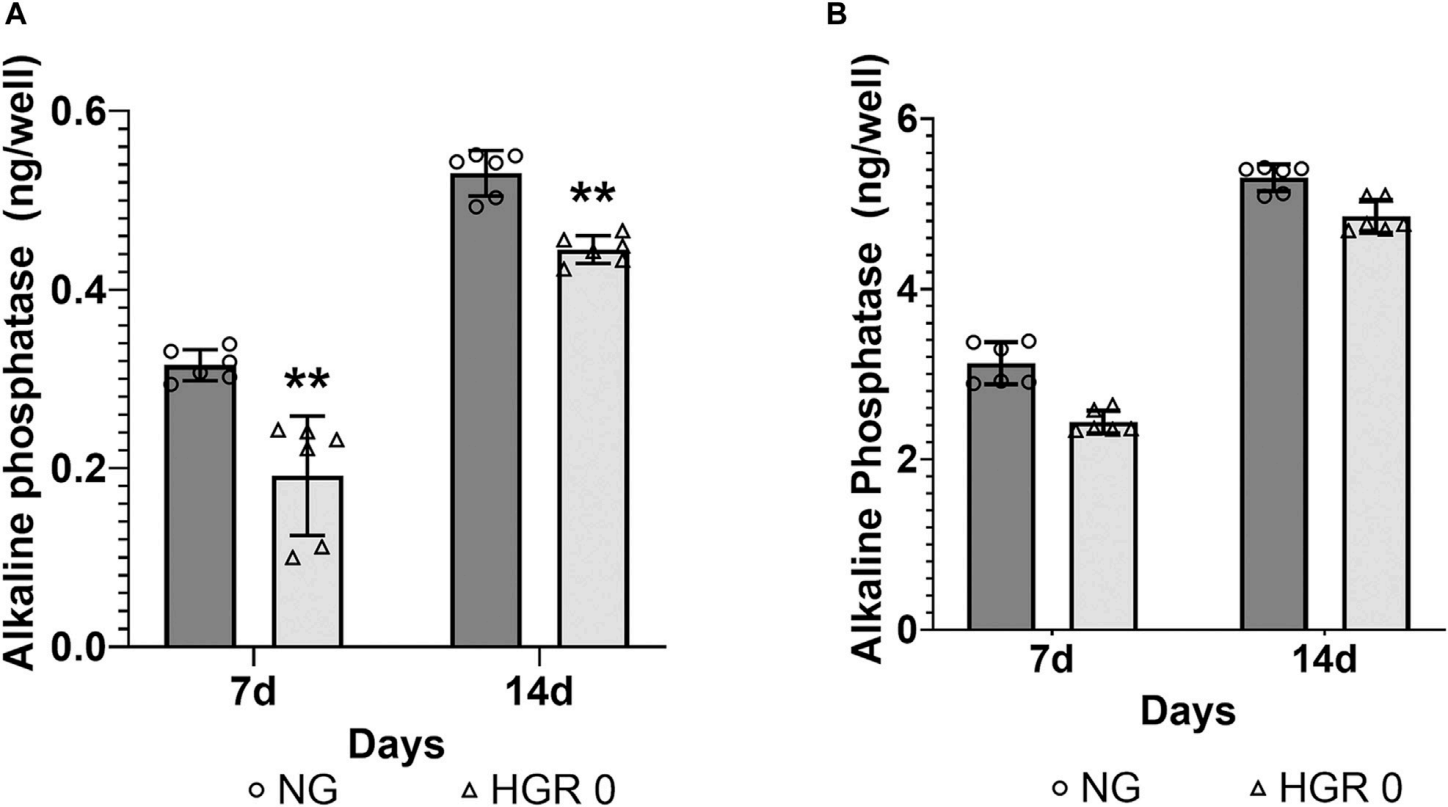
Effect of high glucose on the ALP activity of MC3T3-E1 **(A)** and hPDLF cells **(B)**. ALP activity was evaluated on days 7 and 14 after treatment. In MC3T3-E1 cells, the ALP activity significantly decreased in the HGR 0 group than in the NG group (NG) at all time points (*p <* 0.01) **(A)**. The results indicated that in hPDLF cells, high glucose led to decreased ALP activity, although the difference was not statistically significant **(B)**. It can be observed that the MC3T3-E1 cells were more negatively affected by high glucose than the hPDLF cells. The experiments were conducted independently in triplicate.

The proliferation rates of MC3T3-E1 and hPDLF cells were measured at different time points (days 1, 3, 5, and 7) via CCK-8 assay (Figure 1). In the initial analysis comparing normal and high-glucose conditions, the MC3T3-E1 cells exhibited a significantly lower proliferation rate in the HGR 0 groups than in the NG group on all examined days (*p* < 0.01) (Figure 1A). It was found that the proliferation rate of hPDLF cells was significantly lower in the high-glucose group than in the NG group specifically on day 5 (*p* < 0.01). Although no statistically significant differences were observed at other time points, the proliferation rate decreased in response to high glucose (Figure 1B). These results suggest that both the MC3T3-E1 and hPDLF cells are negatively affected by high glucose, leading to decreased proliferation rates. Notably, the MC3T3-E1 cells were more adversely impacted by high glucose than hPDLF cells. To assess the impact of high glucose on osteogenic differentiation, ALP assay was employed on days 7 and 14 (Figure 2). The MC3T3-E1 cells in the HGR 0 group showed significantly reduced ALP activity (*p* < 0.01) compared with the NG group at all examined time points (Figure 2A). Meanwhile, the hPDLF cells, despite showing a decreasing trend in ALP activity under the high-glucose condition, did not show statistical significance (Figure 2B). This suggests that high glucose exerts a suppressive effect on cellular activity and osteoblast differentiation, particularly in MC3T3-E1 cells. Consequently, MC3T3-E1 cells seem to be more adversely affected by high glucose than hPDLF cells.

### Effect of RSV on cell proliferation and ALP activity under high-glucose conditions

**FIGURE 3:**
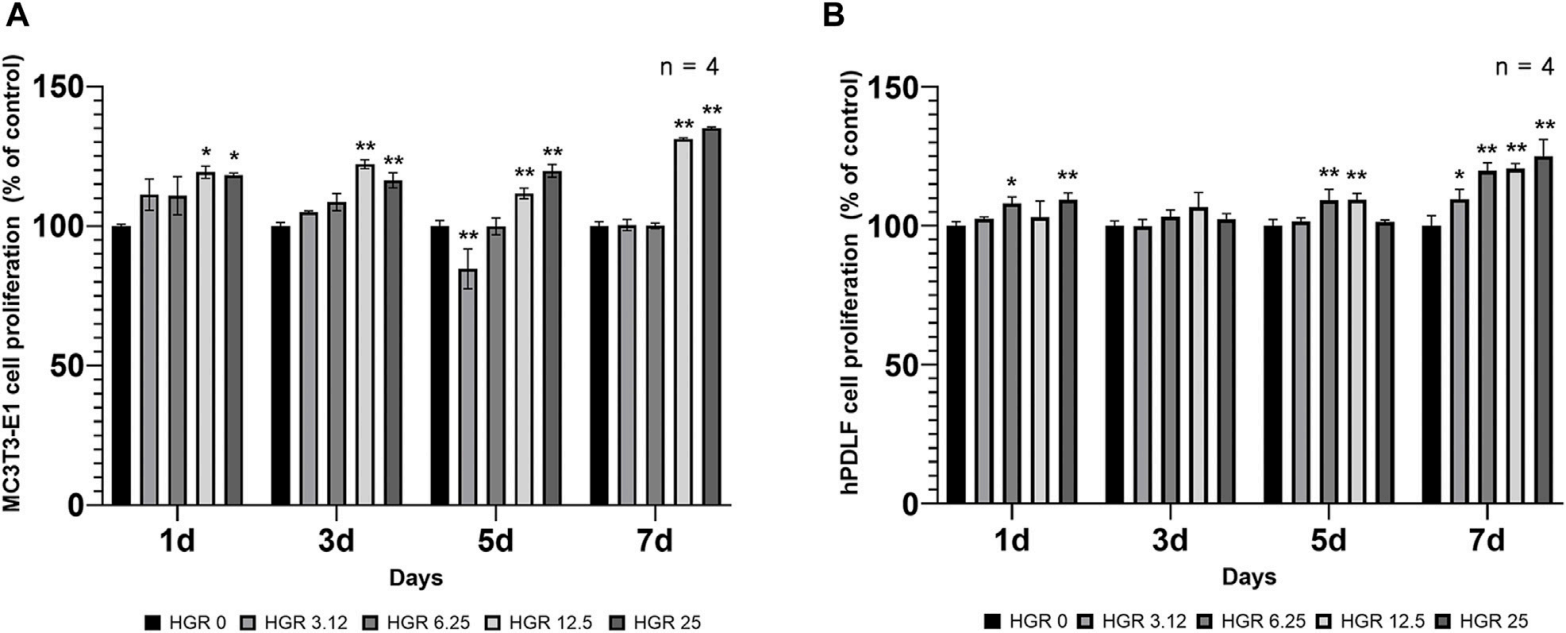
Effect of various RSV concentrations on cell proliferation under high-glucose conditions. In MC3T3-E1 and hPDLF cells, the proliferation pattern was examined at days 1 and 3. The HGR 12.5 and HGR 25 groups showed a significantly increased cell proliferation rate compared to the HGR 0 group at all time points in MC3T3-E1 cells (*p* < 0.05 or *p* < 0.01) **(A)**. In hPDLF cells, the HGR 25 group exhibited the highest proliferation rate on days 1, which was statistically significant compared with that of the HGR 0 group at those specific time points (*p* < 0.05 or *p* < 0.01) **(B)**. The proliferation rate was found to be stimulated by RSV in both cells, but MC3T3-E1 cells may play a more pivotal role in the early stages of bone regeneration than hPDLF cells. To provide clarity and ensure consistency in the nomenclature, the groups were classified as follows: 1) high glucose with 0-μM RSV (HGR 0), 2) high glucose with 3.12-μM RSV (HGR 3.12), 3) high glucose with 6.25-μM RSV (HGR 6.25), 4) high glucose with 12.5-μM RSV (HGR 12.5), and 5) high glucose with 25-μM RSV (HGR 25). The raw data corresponding to the above results had been attached as Supplementary Material S4. *: Statistically significant differences compared with the HGR 0 group at each time point (*p* < 0.05). **: Statistically significant differences compared with the HGR 0 group at each time point (*p* < 0.01).

**FIGURE 4:**
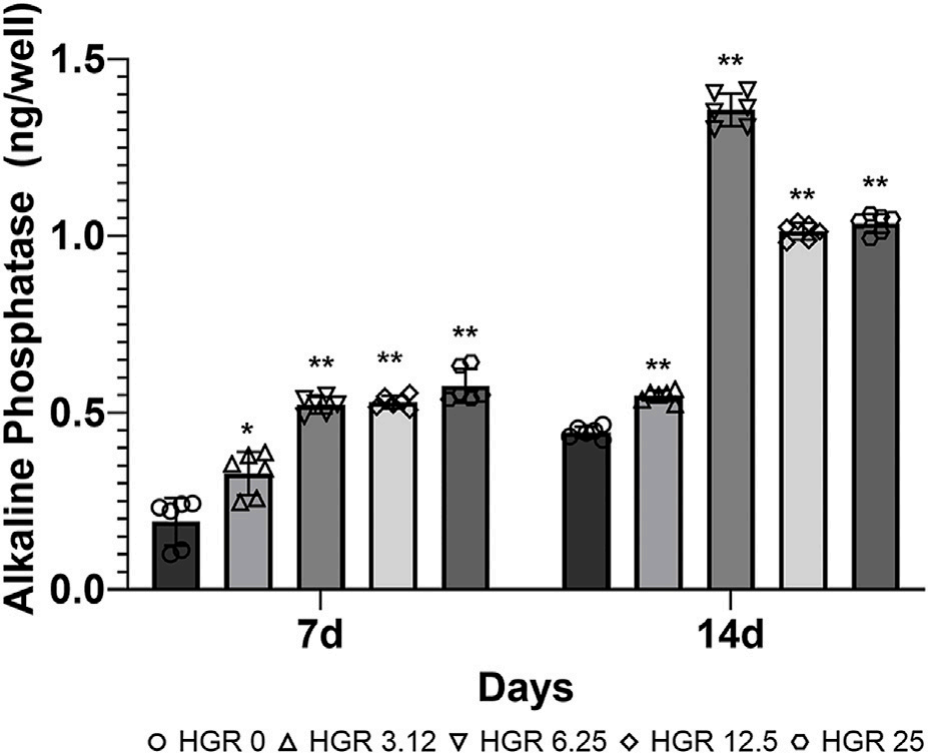
Effect of various RSV concentrations on the ALP activity of MC3T3-E1 cells under high-glucose conditions. The ALP activity in MC3T3-E1 cells was assessed using an ALP assay kit on days 7 and 14 after treatment with various RSV concentrations. The ALP activity rate was significantly higher compared with the HGR 0 group on day 7 at all RSV concentrations (*p* < 0.05 or *p* < 0.01). On day 14, a pattern was observed where the ALP levels increased according to RSV concentration in all groups except for the HGR 6.25 group (*p* < 0.01). Among all groups, it was evident that the HGR 25 group exhibited the most pronounced effect.

We measured the proliferation rate of MC3T3-E1 and hPDLF cells under high-glucose conditions in the presence of different RSV concentrations (Figure 3). We examined the proliferation of MC3T3-E1 cells on days 1 and 3 (Figure 3A). The HGR 12.5 and HGR 25 groups exhibited a significantly increased rate of cell proliferation compared with the HGR 0 group on all time points (*p* < 0.05 or *p* < 0.01). On the other hand, the proliferation rate of hPDLF cells showed a significant increase at a specific concentration on day 1, but no significant results were observed on day 3 (Figure 3B). On day 1, a significant increase was seen in the HGR 6.25 group (*p* < 0.05) and the HGR 25 group (*p* < 0.01), and these results show that RSV has a more positive effect on MC3T3-E1 cells than on hPDLF cells. Unlike hPDLF cells, MC3T3-E1 cells showed consistent responsiveness to RSV against high glucose during all periods examined. Based on these findings, it can be inferred that osteoblasts might play a more pivotal role in early-stage bone regeneration than hPDLF cells. Thus, considering their vulnerability to the detrimental effects of high glucose, we aimed to determine how RSV influences the ALP activity of deteriorated MC3T3-E1 cells. In this study, we examined the ALP activity of MC3T3-E1 cells on days 7 and 14 after treatment with various RSV concentrations using an ALP assay kit (Figure 4). Throughout the entire experimental period (days 7 and 14), all RSV concentrations significantly increased in the ALP activity compared with the HGR 0 group (*p* < 0.05 or *p* < 0.01). With the exception of the HGR 6.25 group on day 14, an increasing trend of the ALP activity was observed across all other groups in response to the RSV concentrations. It was noticeable that the most significant improvement occurred in the HGR 25 group.

### Altered gene expression levels of MC3T3-E1 cells after RSV treatment under high-glucose conditions

**FIGURE 5:**
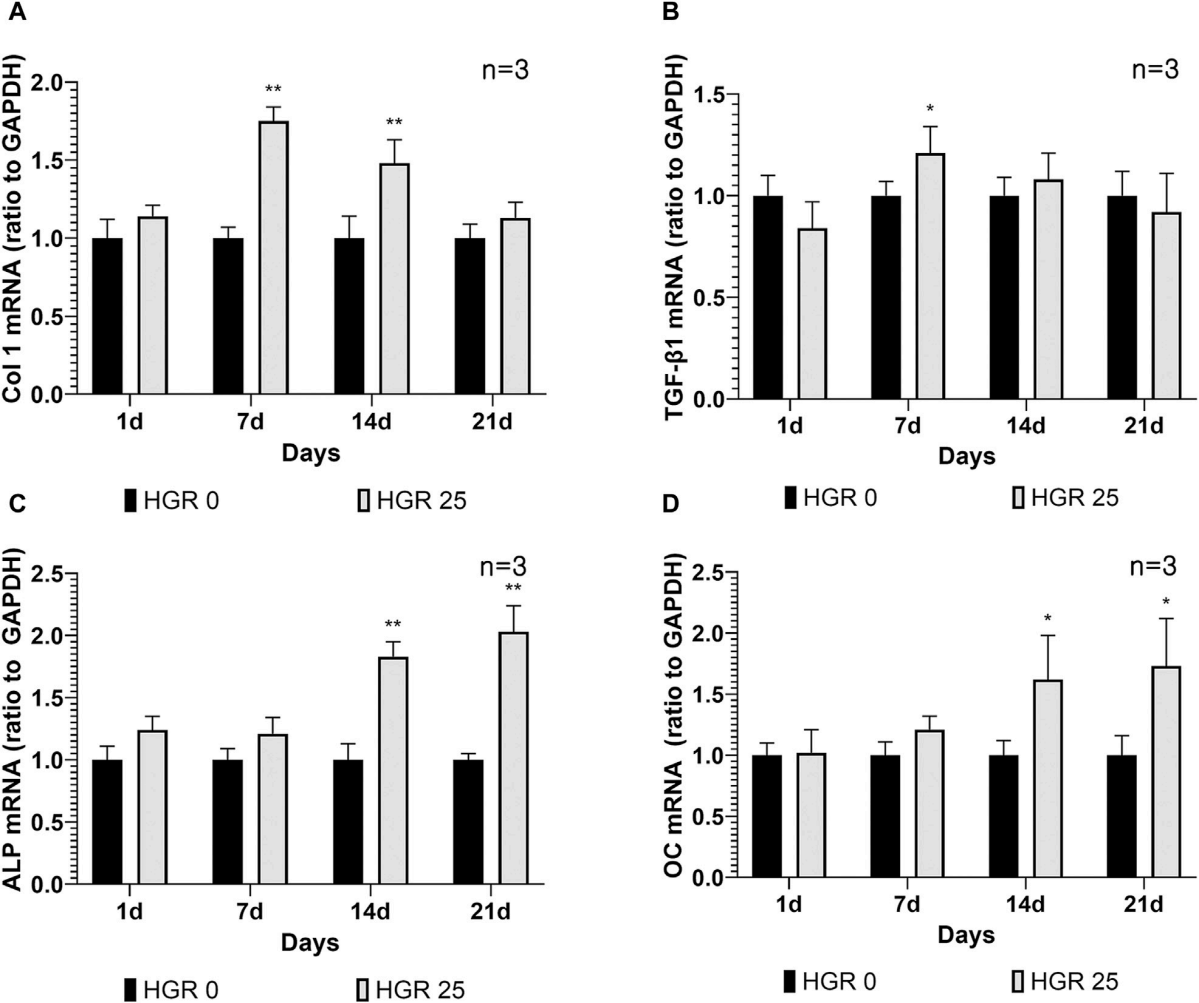
Effect of resveratrol on osteogenesis-related genes of MC3T3-E1 cells under high-glucose conditions **(A–D)**. The expression patterns of key genes including Col 1, TGF-β1, ALP, and OC in MC3T3-E1 cells were analyzed via RT-PCR analysis. The results of the before mentioned experiments indicated that 25-μM RSV was the most effective concentration. On day 7, the HGR 25 group exhibited higher expressions of Col 1 and TGF-β1 than the HGR 0 group (*p* < 0.05 or *p* < 0.01) **(A,B)**. Furthermore, on days 7 and 14, the HGR 25 group exhibited higher expressions of ALP and OC than the HGR 0 group (*p* < 0.05 or *p* < 0.01) **(C,D)**. The raw data corresponding to the above results had been attached as Supplementary Material S5. *: Statistically significant differences compared with the HGR 0 group at each time point (*p* < 0.05). **: Statistically significant differences compared with the HGR 0 group at each time point (*p* < 0.01).

We investigated the altered expression patterns of key genes, including Col 1, TGF-β1, ALP, and OC, in MC3T3-E1 cells via RT-PCR analysis (Figure 5). The analysis was conducted under the influence of high-glucose conditions with administration of RSV to MC3T3-E1 cells. Among all the RSV concentrations, 25 μM was determined to be the most effective, as it yielded the most favorable outcomes in terms of both cell viability and ALP activity. Our assessment encompassed multiple time points: days 1, 7, 14, and 21. Although no substantial increase was observed in the expression levels of all genes on day 1, a significant increase in Col 1 expression became apparent on day 7 (*p* < 0.01) (Figure 5A). This observation was reflected in the significant upregulation of TGF-β1 expression, indicating the active roles of these genes during the initial phases of osteoblast differentiation (*p* < 0.05) (Figure 5B). As we progressed to day 14, the pattern remained consistent for Col 1, exhibiting a persistent and statistically significant increase, albeit with a gradual decline compared with the notable peak observed on day 7. During this period, there was a discernible increase in ALP activity, along with a significant elevation in the expression level of OC, a pivotal marker denoting advanced osteoblast differentiation (*p* < 0.01) (Figure 5C). Remarkably, OC exhibited its most pronounced elevation on day 21 (*p* < 0.05) (Figure 5D). This observation underscores the progressive nature of differentiation and the culmination of osteoblastic activities, as indicated by the increase in late-stage markers.

### Experimentally streptozotocin-induced diabetes mellitus in mice

In our animal study, we induced diabetes mellitus in C57BL-6 male mice using to streptozotocin to investigate the effects of high glucose levels. And then, we compared the diabetic group to a control group with normal glucose levels to assess the impact of high glucose on our experimental outcomes (Supplementary Material S3). After 7 days of treatment, we observed a significant enhancement in the formation of newly mineralized tissue, as evidenced by the intensified blue coloration of collagen, in the normal group. In contrast, the high-glucose group exhibited bone formation primarily in the apical region. Judging from these results, the experimental group, comprising mice with streptozotocin-induced DM, demonstrated reduced bone volume and maturation compared to the normal group. Moreover, in the high-glucose group, the bone healing process was predominantly characterized by the presence of woven bone, whereas the normal group exhibited a more robust healing response with layers of lamellar bone. Additionally, the marginal soft tissue in the normal group exhibited a well-organized fibrous connective tissue, which was lined with keratinized epithelium, indicating a healthier healing environment.

### Effect of RSV on the bone-healing capacity in high-glucose conditions

**FIGURE 6:**
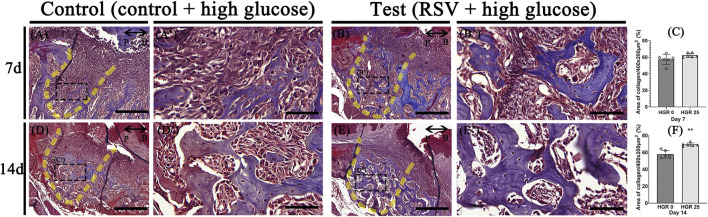
Representative photomicrographs illustrating the histological findings in the high-glucose group **(A,D)** and resveratrol with high-glucose group **(B,E)**. On day 7, the experimental groups exhibit well-developed and calcified bone tissues with dense collagen and a higher density of blood vessel-like structures compared to the control group **(A,A’,B,B’)**. On day 14, both groups show mature bone tissues, but the experimental group exhibit more pronounced bony structures within the tooth socket **(D,D’,E,E’)**. Statistical analysis for newly synthesized collage in the tooth socket **(C,F)**. The solid boxes depict higher magnification view **(A’,B’,D’,E’)**. P, palatal and B, buccal. Dotted line demarcates the root region of the socket. Scale bars denoted 200 μm **(A,B,D,E)** and 50 μm **(A’,B’,D’,E’)**.

We evaluated the impact of RSV on bone healing in mice with induced periodontitis and diabetes by applying 25 μM of RSV (mixed with Pluronic F-127) to the extraction socket of the right upper second molar after extraction. The progression of bone formation was then analyzed histologically at specific time points, including days 7, and 14 (Figure 6). At day 7 post-treatment, the experimental group did not exhibit a significant enhance in bone maturation and mineralization compared to the control group (Figures 6A–C). However, the histological analysis revealed a well-developed collagen matrix and an abundance of vascular structures, indicating a robust bone-healing response. In contrast, the control group exhibited less advanced bone formation and a lower density of blood vessels (Figure 6A). At day 14 post-treatment, both groups demonstrated the presence of mature bone tissues. However, it was evident that the experimental group exhibited more prominent and well-developed formation of bony structures within the tooth socket compared to the control group (Figures 6D–F).

### Altered immunostaining patterns of proteins after RSV treatment in high-glucose conditions

**FIGURE 7:**
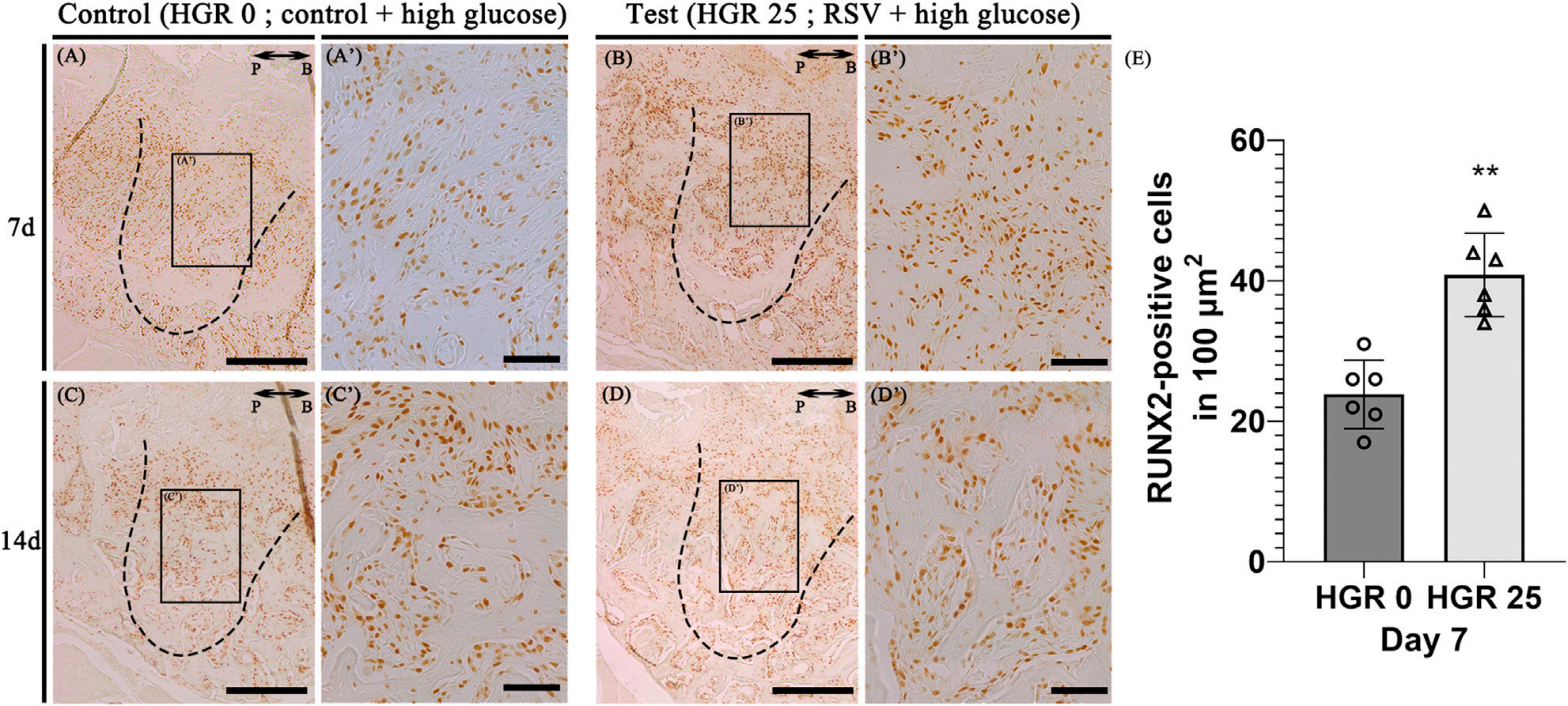
Representative photomicrographs of alveolar tissue sections stained with osteoblast marker (Runt-Related Transcription Factor 2; RUNX2) in the control group **(A,C)** and RSV group **(B,D)**. On days 7 and 14, observable staining of RUNX2 was present in both groups, with a diminishing trend by day 14. RSV group showed increased collagen bundle synthesis with RUNX 2-positive cells compared to the control group on day 7 **(A’, B’)**. The RSV group also show a much stronger localization pattern, especially in the coronal 1/3 compared to that in apical 1/3 and middle regions **(C’, D’)**. Histological analysis using RUNX2 at 7 days **(E)**. The solid boxes depict higher magnification view **(A’–D’)**. P, palatal and B, buccal. Dotted line demarcates the root region of the socket. Scale bars denoted 200 μm **(A–D)** and 50 μm **(A’–D’)**.

Immunostaining for the osteogenic marker (RUNX2) was conducted to elucidate the specific roles of RSV in high-glucose conditions during bone regeneration. We compared the control group (high-glucose group) and the test group (high-glucose group with RSV) on days 7, and 14 (Figure 7). On both days, noticeable staining of RUNX2 was detected in both groups, although the staining appeared to weaken on day 14. RSV-treated specimens exhibited increased synthesis of collagen bundles with RUNX2-positive cells compared to the control group on day 7 (Figures 7A’, B’). On day 14, consistent with histological observations (Figure 6), newly formed bone matrices were evident in both groups (Figures 7C, D).

## Discussion

DM is a highly prevalent disease characterized by elevated glucose levels and various complications. The intricate association between DM and periodontitis is well established (Genco and Borgnakke, 2000; Preshaw and Bissett, 2013; Wu et al., 2015; Wu et al., 2020). DM has been observed to exacerbate the severity of periodontitis and accelerate bone resorption. To verify this, we examined how DM impacts the cells relevant to bone resorption at the cellular level (Figures 1, 2). Our findings indicate that elevated glucose levels have a detrimental effect on both MC3T3-E1 and hPDLF cells, resulting in reduced cell proliferation (Figure 1). MC3T3-E1 cells exhibited a notable decrease in cell proliferation under high-glucose conditions compared with hPDLF cells (Figure 1). Studies have shown that DM aggravates osteoblastic cell apoptosis, leading to decreased osseous coupling, and aggravates loss of osteoblasts and hPDL fibroblasts through increased apoptosis in response to periodontitis (Isaka et al., 2001; Liu et al., 2006; Pacios et al., 2013). Furthermore, several studies also reported a decrease in cell viability and osteoblast and hPDL fibroblast proliferation under a high-glucose condition [(Kim et al., 2006; Kato et al., 2016)]. To gain deeper insights into the intricate effects of high-glucose conditions on cellular physiology, we assessed ALP activity to determine its influence on osteoblast differentiation (Figure 2). ALP activity, recognized as a dependable marker for evaluating osteoblastic cell phenotype, served as a valuable indicator for our investigations. Consistent with the results of previous studies, our findings indicated a reduction in ALP activity in MC3T3-E1 cells exposed to high-glucose conditions (Figure 2A). As previously reported, this reduction in ALP activity suggests inhibited osteogenic differentiation in the presence of high glucose (Dong et al., 2017). Given the well-established connection between oxidative stress and DM, which is known to significantly affect osteoblast differentiation and overall bone metabolism (Mody et al., 2001; Fatokun et al., 2006; Wu et al., 2015), a reduction in ALP activity in osteoblasts due to DM would have detrimental effects on bone metabolism (Mody et al., 2001). These findings highlight the detrimental effects of high glucose on cell proliferation and bone formation.

We confirmed that DM decreased the bone-healing capacity in mouse extraction sockets (Supplementary Material S3). On post-treatment day 7, we observed that the formation of newly mineralized tissue shown by blue-colored collagen with MTC staining was higher in the normal group than the high glucose group. These findings were similar to the results of a previous report by Devlin H et al., (Devlin et al., 1996), which suggested that in DM, the formation of a collagenous framework in the tooth extraction socket was inhibited, resulting in delayed healing and alveolar bone resorption. In addition, several studies reported that Type 1 DM increased the severity of alveolar bone loss induced by periodontitis in rats (Kim et al., 2014a; Kim et al., 2014b). High glucose mice also showed higher infiltration of inflammatory cells in the junctional epithelium and connective tissue compared to the normal glucose group. This result would imply that high glucose levels increase inflammation.

RSV has been extensively studied and validated as a potential therapeutic agent for periodontitis, an inflammatory condition caused by bacteria and characterized by alveolar bone loss. This includes both *in vitro* and *in vivo* experiments, demonstrating the facilitating effects on osteoblast differentiation at the cellular and tissue levels (Bhattarai et al., 2016a; Min et al., 2020). Furthermore, animal studies have demonstrated the efficacy of RSV in alveolar bone regeneration, reaffirming its potential as a promising treatment agent for periodontal disease (Ribeiro et al., 2017). Nonetheless, despite the promising potential of RSV as a treatment for periodontitis, there are several challenges in its clinical application. Notably, robust evidence of its efficacy in cases where alveolar bone loss is exacerbated by diabetes is scarce. Thus, as the impact of RSV on periodontal regeneration has been previously investigated, we sought to delve into its effects specifically under high-glucose conditions. To conduct this analysis, we used bone-forming cells, encompassing both MC3T3-E1 and hPDLF cells (Figure 3). The findings indicated that cell proliferation rates increased at specific RSV concentrations under high-glucose conditions. Similar observations have been reported in previous studies (Mizutani et al., 1998; Min et al., 2020), where RSV was found to stimulate MC3T3-E1 and hPDLF cells proliferation. The results suggest that RSV exerts a more immediate effect on osteoblasts than on hPDLF cells under high-glucose conditions, with a significant impact observed in the early stages (Figure 3). However, as time progresses, the influence of RSV seems to be more pronounced on hPDLF cells. Based on these observations, it can be concluded that RSV acts more swiftly on deteriorated osteoblasts affected by high-glucose conditions. This study also demonstrated that RSV treatment increased ALP activity (Figure 4). Similar to the proliferation rate, the ALP activity decreased at high RSV concentrations. RSV has shown promising potential in stimulating proliferation and differentiation of MC3T3-E1 cells involved in bone formation and regeneration. A previous study conducted randomized controlled trials and reported that RSV supplementation was correlated with increased levels of bone ALP, suggesting enhanced bone formation (Ornstrup et al., 2014). Another study investigated the effects of RSV on the differentiation of MC3T3-E1 cells *in vitro* and observed a direct stimulatory effect on bone formation (Mizutani et al., 1998). These findings support the notion that RSV exerts beneficial effects on bone health and suggests its potential as a therapeutic agent for promoting bone regeneration and treating bone-related conditions.

A notable aspect of this study is the observation that under high-glucose conditions, osteoblasts were more adversely affected than hPDLF cells (Figures 1, 2). Osteoblasts and hPDL fibroblasts are both essential components of the periodontal tissue and play crucial roles in maintaining the periodontium. However, the observed disparities could likely stem from differences in the sensitivity of these two cell types to high-glucose levels. The lack of significant findings about hPDL fibroblasts might be attributed to the relatively short duration of the experiments. Furthermore, a significant observation in our study was that the positive effects of RSV, particularly under high-glucose conditions, appeared at an earlier stage in MC3T3-E1 cells compared with hPDLF cells (Figure 3). This suggests that RSV has a quicker and more pronounced impact on MC3T3-E1 cells, which are osteoblastic cells, in a hyperglycemic environment (Mizutani et al., 1998; Dai et al., 2007; Min et al., 2020). This distinction in the timing and extent of the effects of RSV between these two cell types suggests that it could potentially play a more rapid and robust role in countering the adverse effects of high glucose on osteoblastic cells, possibly contributing to bone homeostasis restoration. The exact reasons for these differences are unknown, but they could potentially be attributed to the properties of RSV, which would include its ability to directly neutralize ROS (Truong et al., 2018). This distinction in how the two cell types respond to RSV might be associated with their varying reactions as an antioxidant. Additionally, osteoblasts are regulated by multiple signaling pathways, including the SMAD 2/3-independent signaling pathways by which the bone morphogenetic proteins from the TGF-β superfamily regulated cell proliferation and differentiation (Boby et al., 2021). Our study showed that the expression levels of TGF-β1 in MC3T3-E1 cells progressively increased (Figure 5B). The intrinsic differences in mechanisms might have accelerated the differentiation of MC3T3-E1 cells compared to hPDLF cells. Based on our results, the further research is needed to explore these distinctions more deeply.

In the present study, we investigated the expression patterns of key markers involved in bone formation and mineralization, such as Col 1, TGF-β1, ALP, and OC, in MC3T3-E1 cells (Figure 5). Col 1, as a major component of the bone extracellular matrix, plays a critical role in the organization and mineralization of the bone matrix. Its expression levels were assessed via RT-PRC at different time points (days 1, 7, 14, and 21). This analysis provides insights into the dynamic regulation of Col 1 expression during bone formation and highlights its importance in bone matrix integrity and mineralization (Terajima et al., 2014). MC3T3-E1 cells exhibited high levels of Col 1 expression during the early stages and showed peak expression on day 7. TGF-β1, a key factor involved in extracellular matrix formation, plays a critical role in bone cell differentiation (Bismar et al., 1999). Its involvement in collagen production is necessary for the subsequent mineralization process, highlighting the importance of TGF-β1 and Col 1 in the early and middle stages of bone formation (Choi et al., 1996). Furthermore, we assessed the activity of ALP, an early marker of osteoblast differentiation (Ortiz-Vigon et al., 2017), and found a significant increase in ALP activity in MC3T3-E1 cells after 14 days of RSV treatment. In addition, the expression of OC, a late-stage marker of osteoblast differentiation (Li et al., 2017), was significantly elevated on days 14 and 21. These findings are consistent with those of previous studies by Choi et al. (Choi et al., 1996). and Min et al. (Min et al., 2020), supporting the notion that RSV treatment enhances the expression of bone-related proteins such as ALP, BMP-2, BMP-4, and OC. Overall, our results indicate that the application of RSV to both cells under high-glucose conditions may promote bone formation and maturation by modulating these bone-related proteins.

We also evaluated to validate bone healing capacity of RSV in high-glucose conditions through streptozotocin-indued DM mice model. To induce DM in mice, we injected streptozotocin (STZ) i.p. at a dose of 40 mg/kg. STZ is widely used to induce DM in experimental animals by causing the selective destruction of pancreatic β-islet cells (Kim and Kim, 2006). There is a strong influence of gender on DM induction because females are resistant to the effects of low-dose STZ (Kolb, 1987). Therefore, we only induced DM in male mice in this study. The common protocol for inducing DM in mice is to produce a type 1 DM model with the necrosis of islet β-cells induced by STZ (Furman, 2015). However, type 2 DM accounts of 90% of the diabetes cases globally (Zimmet et al., 2001), which must be a consideration in the analysis of these studies. To mimic the periodontitis-induced extraction socket, a model of ligature-induced periodontitis in mice was used. The ligatures were thought to facilitate the local accumulation of bacteria and enhance inflammation and bone loss (Graves et al., 2008). In this study, the maxillary right second molars of the mice were tied for 5 days. The rodent model has the advantage of mice having a molar structure similar to humans (Oz and Puleo, 2011). Some studies reported that the bone levels on the buccal and palatal sides were significantly lower than the control after 5 days (Adhikari et al., 2019). These results suggested that 5 days are adequate to assess the inflammation and bone loss and, quite likely, represent a period of disease progression (Abe and Hajishengallis, 2013). Thus, we decided to maintain the ligation for 5 days in this study.

RSV was applied to facilitate osteogenesis and its effects were compared with the control group at days 7, and 14 (Figure 6). These results were similar to previous findings, which reported that RSV induced bone formation by stimulating proliferation and differentiation of osteoblasts (Durbin et al., 2014). In some studies, RSV was shown to decrease the inflammatory response in diabetic periodontitis by down-regulating TLR4 expression (Zhen et al., 2015). It is thought that the positive effects of RSV on bone formation could result from anti-inflammatory activity, and it has also been shown to have a structural similarity to several hormones that positively influence bone formation (Bhattarai et al., 2016b). In addition, cuboidal cells are clearly visible at the bone surface margin in RSV group. Therefore, RSV may stimulate the osteoblast and osteoblast precursor cells in the extraction socket and contribute to bone regeneration. Furthermore, the intensified localization pattern of the pre-osteoblastic marker, RUNX2, on day 7 in the experimental group suggests that RSV may facilitate osteogenesis (Figure 7). On day 14, as evident from the Masson’s trichrome staining (Figure 6), where significant bone formation is observed, there appeared to be no substantial difference in RUNX2 expression. Our findings imply that RSV modulates the proliferation and differentiation of osteoblasts during bone healing and regeneration (Min et al., 2020).

These findings suggest the potential application of RSV in clinical settings. Particularly, from the standpoint of bone regeneration, RSV is being considered as a potential therapeutic agent for treating osteoporosis (Mobasheri and Shakibaei, 2013). In dentistry, RSV has the potential to induce alveolar bone regeneration. Combining RSV with periodontal therapy may enhance clinical parameters and reduce inflammation in patients with periodontitis (Nikniaz et al., 2023). However, it is noteworthy that research on delivery systems and scaffolds is necessary for clinical applications. Studies on localized RSV application have also been conducted. In a study by Uysal et al., the effects of locally applied RSV on bone formation in rats were evaluated in response to the expansion of the interpremaxillary upper incisors (Uysal et al., 2011). However, the poor bioavailability of RSV is a significant drawback. Suitable scaffolds are currently being developed. It is anticipated that such advances will further facilitate the clinical application of RSV. Notably, RSV is known to exert positive effects on diabetes, and based on this study, it may further facilitate the regeneration of periodontal tissue loss exacerbated by diabetes.

Taken together, our results indicate that elevated glucose levels can exert detrimental effects on cellular activity and capacity for bone healing. However, RSV has been found to enhance the physiological functions of both MC3T3-E1 and hPDLF cells, suggesting its potential role in functional restoration in periodontal regeneration. Furthermore, it was observed that RSV would facilitate in the formation of bone, exacerbated DM and periodontitis. Nonetheless, further research is warranted to investigate the more positive effects on osteoblasts than on hPDL fibroblasts and the earlier onset of these effects.

## Data Availability

The raw data supporting the conclusion of this article will be made available by the authors, without undue reservation.

## Abbreviations

ALP, alkaline phosphatase; α-MEM, alpha-minimum essential media; Col 1, collagen type 1; DM, diabetes mellitus; DMEM, dulbecco’s modified eagle’s medium; EDTA, ethylenediaminetetraacetic acid; ELISA, enzyme-linked immunosorbent assay; FBS, fetal bovine serum; GAPDH, glyceraldehyde-3-phosphate dehydrogenase; hPDLF, human PDL fibroblasts; MTC, masson’s trichrome; OC, osteocalcin; PBS, phosphate-buffered saline; ROS, reactive oxygen species; RSV, resveratrol; RT-PCR, real-time polymerase chain reaction; RUNX2, runt-related transcription factor 2; TGF-β1, transforming growth factor-β1.
