# Supplementary material for: Resveratrol facilitates bone formation in high-glucose conditions
Source: Front Physiol. 2024 Apr 19;15:1347756. doi: 10.3389/fphys.2024.1347756 (PMC11066205; doi:10.3389/fphys.2024.1347756)
Supplement: Supplementary file 1 [file DataSheet1.docx]

Supplementary Material

The effects of resveratrol on facilitating of bone formation in high-glucose conditions

**Sung-Min Hwang**^*^**, Tae-Young Kim, Anna Kim, Yong-Gun Kim, Jin-Woo Park, Jae-Mok Lee, Jae-Young Kim, Jo-Young Suh**

*** Correspondence:**

Jo-Young Suh, DDS, PhD

Department of Periodontology, School of Dentistry, IHBR, Kyungpook National University, 2177, Dalgubeol-daero, Jung-gu, Daegu 41940, Korea

Tel: 82-53-600-7521

E-mail: jysuh@knu.ac.kr

Jae-Young Kim, PhD

Department of Biochemistry, School of Dentistry, IHBR, Kyungpook National University, 2177, Dalgubeol-daero, Jung-gu, Daegu 41940, Korea

Tel: 82-53-420-4999

Fax: 82-53-421-4276

E-mail: [jykim91@knu.ac.kr](mailto:jykim91@knu.ac.kr)

# Supplementary Figures and Tables

## Supplementary File 1

| **Groups** | **Treatments** | **Remark** |
| --- | --- | --- |
| 1 | Normal glucose conditions | NG |
| 2 | High-glucose conditions only | HGR 0 |
| 3 | 3.12-μM RSV with high-glucose conditions | HGR 3.12 |
| 4 | 6.25-μM RSV with high-glucose conditions | HGR 6.25 |
| 5 | 12.5-μM RSV with high-glucose conditions | HGR 12.5 |
| 6 | 25-μM RSV with high-glucose conditions | HGR 25 |

## To facilitate classification, the groups were labeled as follows:

## Supplementary File 2


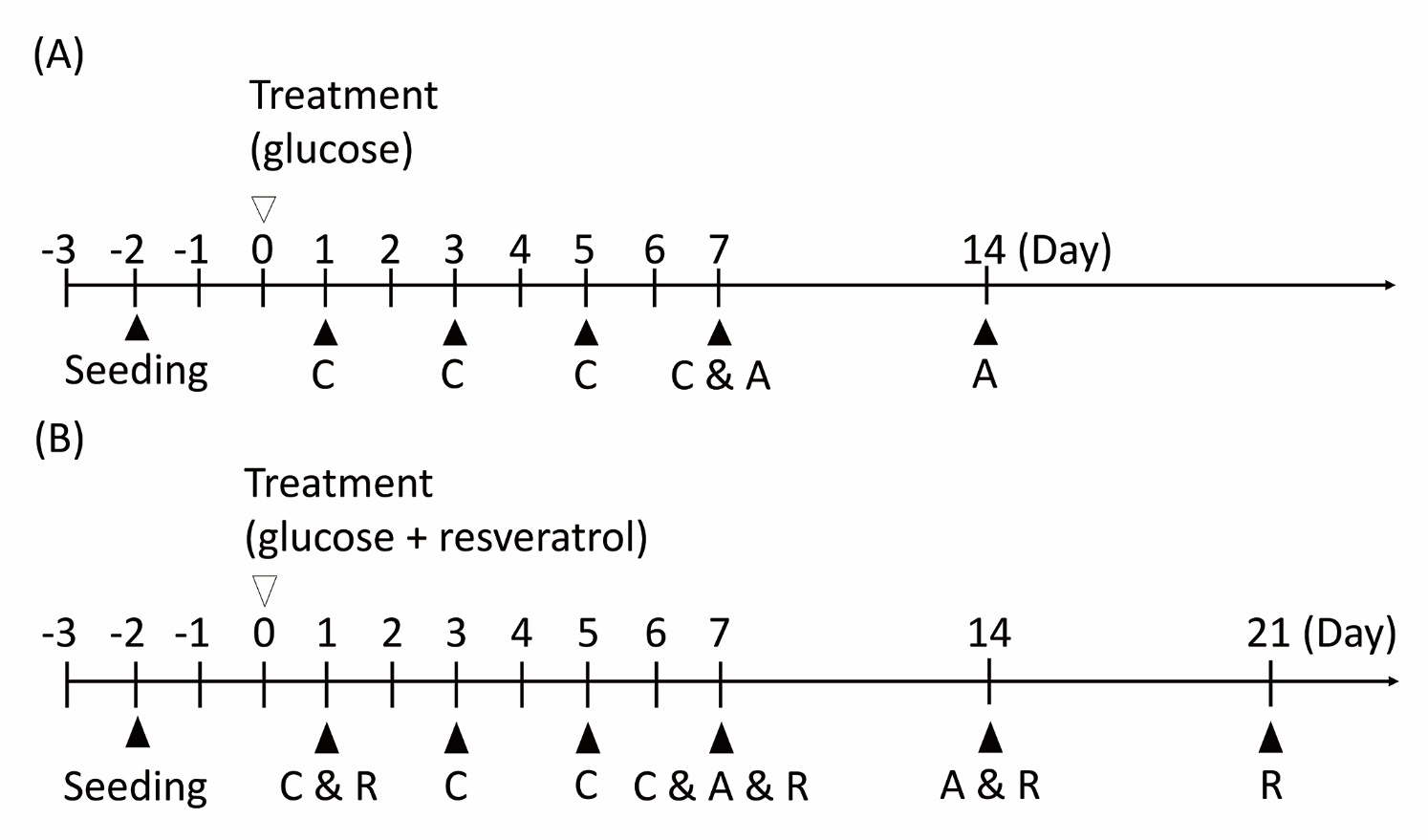


**Schematic diagram of the in vitro cell culture and treatment paradigm.** (A) Establishment of cell cultivation in high-glucose conditions. (B) Evaluation of resveratrol in high-glucose conditions. *A: ALP, C: CCK-8, R: RT-PCR*

## Supplementary File 3


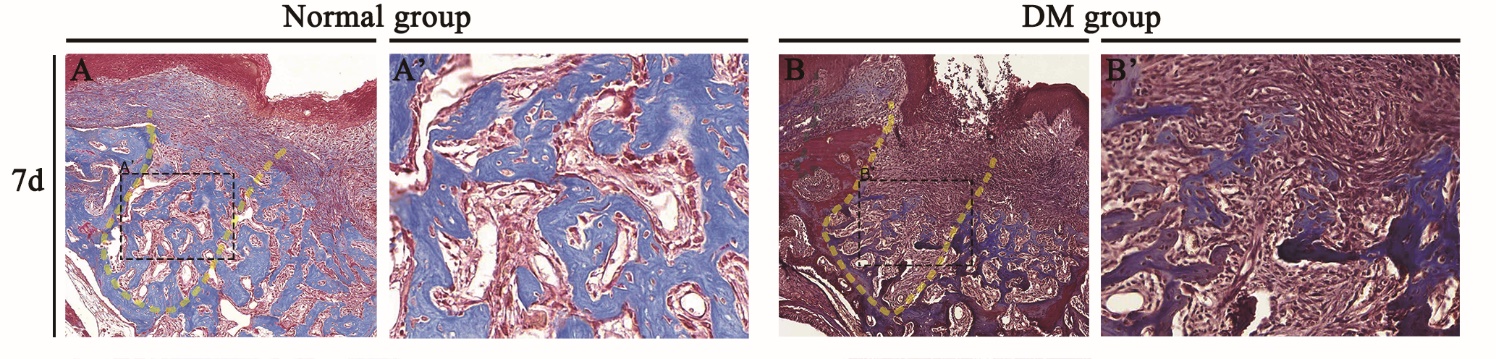


**Effect of high-glucose on the bone-healing capacity *in vivo*.** To assess the impact of high-glucose on bone-healing capacity, an animal model using C57BL-6 male mice was employed. DM was induced in the mice using streptozotocin, and their bone healing was compared to a control group with normal glucose levels. On day 7, we observed the formation of newly mineralized tissue, indicated by the blue-colored collagen. In the normal-glucose group (A, A’), a higher amount of blue-colored collagen was observed, signifying increased formation of newly mineralized tissue. Conversely, in the high-glucose group (B, B’), a lower amount of blue-colored collagen was observed. These findings confirm that the normal-glucose group exhibited enhanced bone formation compared to the high-glucose group. Scale bars denoted 200 μm (A, B) and 60 μm (A', B’).

## Supplementary File 4


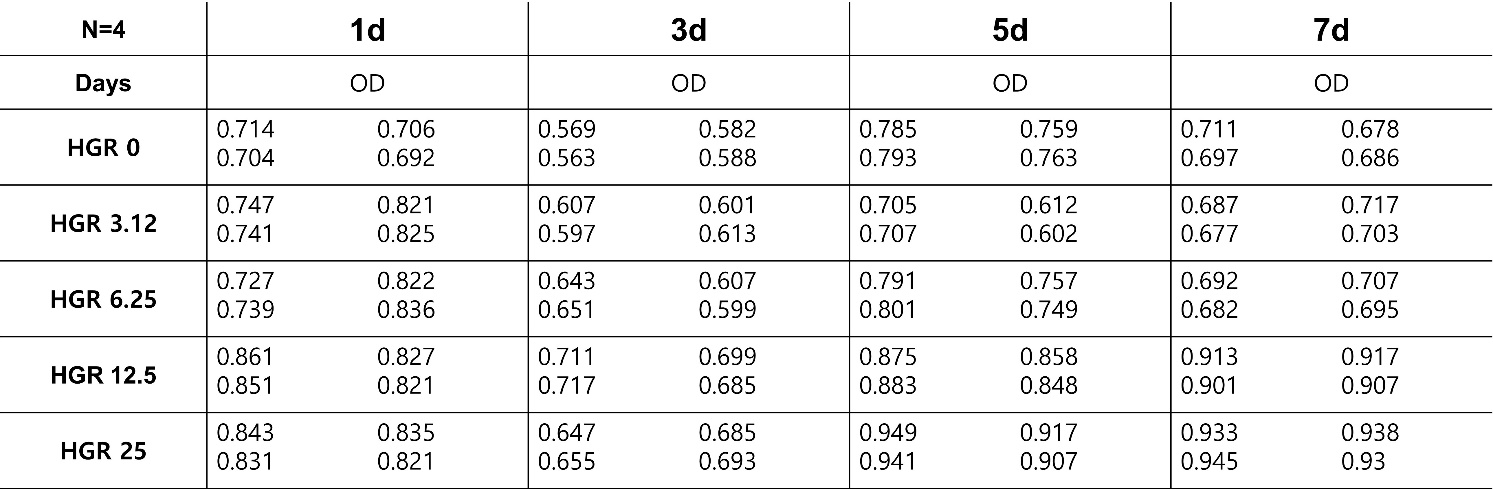


Raw data of cell proliferation for assessing effect of various RSV concentrations under high-glucose conditions

## Supplementary File 5


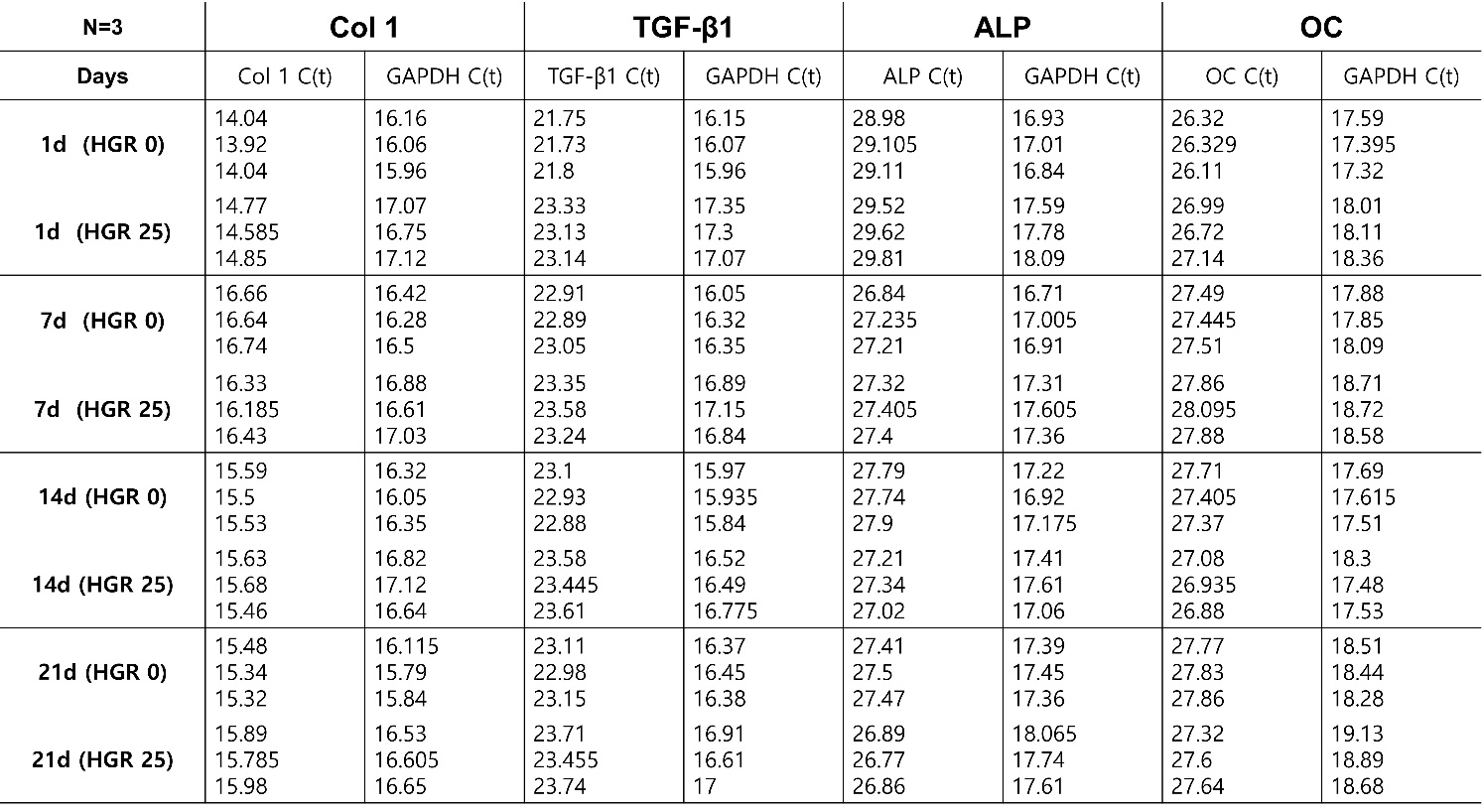


Raw data of qRT-PCR for assessing effect of RSV under high-glucose conditions
